# Access to maternity waiting home services and its determinants among women in Ethiopia: systematic reviews and meta-analysis

**DOI:** 10.3389/fgwh.2024.1423639

**Published:** 2024-12-09

**Authors:** Kassahun Tafesse Hidoto, Kassa Daka Gidebo, Mengistu Meskele Koyira, Maria Luisa Guillén Domínguez

**Affiliations:** ^1^ School of Public Health, College of Health Sciences and Medicine, Wolaita Sodo Univerisity, Wolaita Sodo, Ethiopia; ^2^ Department of Preventive Medicine and Public Health, Food Science, Toxicology, and Legal Medicine, University of Valencia, Valencia, Spain

**Keywords:** women, maternity waiting home, utilization, systematic review, meta-analysis, Ethiopia

## Abstract

**Introduction:**

Globally, maternal mortality is a major public health problem mainly due to a lack of access to skilled care during childbirth. Maternity waiting homes (MWHs) play a critical role in accessing emergency obstetric care for pregnant women during childbirth. However, available studies show inconsistent findings about women's utilization of maternity waiting homes. Therefore, the aim of this review was to identify the pooled prevalence of women's utilization of maternity waiting homes and its associated factors in Ethiopia.

**Method:**

We used the PRISMA guidelines to report the review. We searched for potentially eligible studies in Google Scholar, PubMed, Cochrane Library, and Google using Medical Subject Heading terms and keywords. The retrieved articles were screened and assessed for quality. The heterogeneity across studies was checked using Cochran's *Q* test and *I*^2^ statistics. The pooled levels of women's utilization and associated factors were analyzed using meta-analysis. The publication bias was measured using the funnel plot and Egger's test. The subgroup analysis and sensitivity analysis were carried out to identify the studies with high effects.

**Results:**

A total of 11 eligible studies with 11,784 study participants were included. The utilization of MWHs was in the range of 7%–42.5%. The pooled estimate of women's utilization of maternity waiting homes was 22.49%. Factors associated with utilization included women's decision-making power, access to transport, walking distance to the nearest facility, and having a companion.

**Conclusion and recommendation:**

The overall prevalence of maternity waiting home utilization in Ethiopia is low, with significant variation across regions. Health sector program administrators should focus on both the consumer and healthcare system.

**Systematic Review Registration:**

The review protocol was registered in PROSPERO (number CRD42021243526).

## Introduction

Maternal mortality is still a major public health problem in many sub-Saharan countries, including Ethiopia (1). To alleviate this problem; maternity waiting homes (MWHs) were introduced in developing countries in the early 20th century, mainly in rural areas where women lack access to maternal healthcare facilities (2).

Women's health is a fundamental issue that impacts not only women but also their families, society, and the state (3). Maternal health is an important principle for healthcare quality and planning, which is also central to the Sustainable Development Goals (SDGs) agenda. This agenda highlights the importance of sustained high concern for maternal and newborn health (MNH) under SDG-3, aiming to reduce maternal mortality to 70 per 100,000 live births by 2030 (4).

The World Health Organization (WHO) recommends that trained professionals attend all childbirths in facilities equipped with emergency obstetric and newborn care to avert maternal and neonatal deaths (5). Delivering in health facilities with the capacity for basic or comprehensive emergency obstetric and neonatal care has shown improved maternal and infant health outcomes (6). The utilization of maternity waiting homes is critical in reducing maternal mortality rates (7, 8). These housing facilities are located near hospitals or health centers to accommodate women in the final weeks of pregnancy, bridging the geographical barriers to healthcare access (9, 10). This strategy is unattainable without evidence-based planning and implementation.

Primary studies conducted in Ethiopia on maternity waiting homes have identified varied levels of utilization and multiple influencing factors (11–21). Key factors affecting MWH utilization include the costs associated with using these facilities, food security within the MWHs, the length of time spent away from family, the presence of pregnancy complications, women's autonomy in decision-making, and the quality of services provided (11–17, 19–21).

These findings are not homogeneous and are highly context-dependent, utilization levels in the range of 7.0%–42.5%.

A systematic review and meta-analysis was conducted to address the lack of pooled evidence on maternity waiting home utilization and its associated factors in Ethiopia. These findings could be helpful to improve strategies and reduce maternal mortality and morbidity.

## Methods and materials

### The review approach and protocol development

We used the Preferred Reporting Items for Systematic Reviews and Meta-Analyses (PRISMA) guidelines to report each section of the review (22). The review protocol was developed and registered in the International Prospective Register of Systematic Reviews (PROSPERO; registration number CRD42021243526).

### Search strategies

The search was conducted between 15 March and 6 October 2021. We searched for potentially eligible primary studies in Google Scholar, PubMed, Cochrane Library, and Google search engines. The search terms within the same concepts were connected with the Boolean operator “OR” and combined with other terms using the Boolean operator “AND.” A combination of keywords and Medical Science Heading (MeSH) terms were also used. The PubMed and Cochrane Library databases were searched using; “utilization” OR “use” AND “maternity waiting home” OR “maternity waiting area” OR “maternity waiting house” AND “associated factors” OR “determinants” OR “factors affecting” AND “Ethiopia.” Similarly, for the Google Scholar and Google search engine databases, we used “maternity waiting homes” AND “Utilization” or “factors affecting” AND “Ethiopia.” Two authors (KH and MD) conducted independent searches.

### Study selection process

All studies retrieved from the databases were exported to the Endnote reference manager software library. Articles published in the English language and conducted in Ethiopia since 2010 were considered eligible for this review. We used the 2010 as the starting point for the studies because the nationwide scale-up of MWHs in Ethiopia began in 2010 per the World Health Organization’s recommendation. After duplicates were removed, studies were screened by reading the titles and abstracts. Any articles that did not fulfill the inclusion criteria were excluded at each level of review.

A complete full-text review was conducted to identify relevant articles addressing the prevalence of maternity waiting home utilization and its associated factors. Quality assessments were conducted using the Joanna Briggs Institute's System for the Unified Management, Assessment, and Review of Information (JBI-SUMARI) premium software, which provides a quality appraisal checklist (23). Studies considered as low risk and scoring 6/9 or higher were deemed eligible and included in the analysis.

### Inclusion and exclusion criteria

#### Inclusion criteria

The inclusion criteria were primary cross-sectional or prevalence studies conducted in Ethiopia that reported the prevalence and/or at least one associated factor with MWH utilization between 2010 and 2021. The review also included gray literature.

#### Exclusion criteria

The exclusion criteria included articles in languages other than English, non-primary studies, studies that provided only titles and/or abstracts, anonymous reports, commentaries, and letters.

### Operational definition

Maternity waiting home utilization refers to a woman self-reporting her admission to a functional MWH in the final weeks of her pregnancy, where she remains until childbirth and for 24 h after delivery (24).

### Data extraction and review process

Before data extraction, the full texts of the selected studies were reviewed to ensure they met the inclusion criteria. Data were then extracted on the following: author(s), year of study, study design, study area, period of study, sample size, prevalence of MWH utilization [estimated magnitude with 95% confidence intervals (CIs)], and factors affecting MWH utilization, including the respective odds ratio (OR).

For studies with missing required variables, the authors were contacted to obtain the necessary data. Studies with complete and clear numerical data were included in the meta-analysis to calculate the pooled prevalence and assess the association of variables. Data extraction was conducted independently by two authors (KH and MD). Any disagreements between the reviewers in the data extraction process were resolved by the third author (KG or MMK) to reach the final decision.

### Methodological quality

The methodological quality of the primary studies was independently assessed by two authors (KH and MD). The critical appraisal quality of each study was conducted using the JBI-SUMARI online-supported software for cross-sectional studies (23). Studies that scored 6/9 criteria and above were included in the analysis.

### Data analysis and presentation

The authors used Microsoft Excel for data entry and STATA version 14 software for data analysis. To determine the pooled prevalence of MWH utilization and identify associated factors, the random-effects model was applied, with effect measures and 95% CIs.

### Publication bias and heterogeneity test

To minimize the risk of bias, the authors applied all the relevant techniques mentioned below. The primary articles were searched both electronically and manually to avoid missing relevant studies. The articles were selected according to the set criteria and objectives. Two authors independently performed the screening and data extraction. Publication bias was assessed visually through a funnel plot graph. Quantitatively, we used Egger's correlation test at a 5% significance level to check for potential publication bias. The sensitivity analysis was carried out using *I*^2^ statistics with a significance level of *p* ≥ 0.05, applying the random-effects model. We also conducted a subgroup analysis to reduce the random discrepancies among the studies by study regions and publication year.

## Result

### Search results

**Figure 1:**
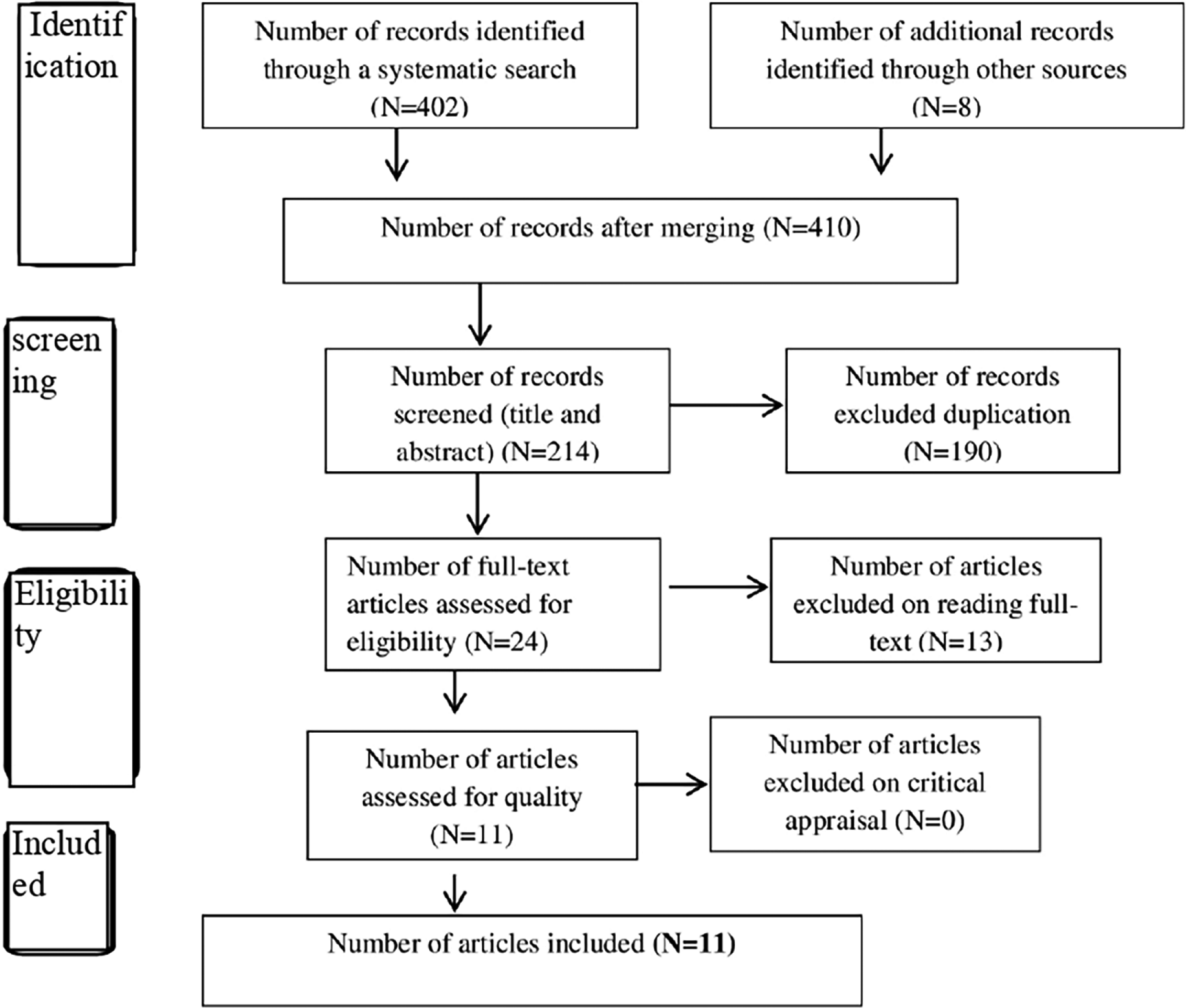
Characteristics of studies searched for utilization of maternity waiting home in Ethiopia, 2021.

We searched for primary studies conducted in Ethiopia and published between 2010 and 2021 for inclusion in this review. A total of 410 citations were found through the electronic database searches and exported to Endnote. After evaluating titles and abstracts, 196 studies were excluded for not meeting the inclusion criteria. Of those 214, 190 duplicates were removed, leaving 214 studies. The 24 full-text studies were then reviewed against the inclusion criteria. Of them, 13 studies were excluded because the full text could not be accessed. Finally, 11 articles were selected for the final systematic review and meta-analysis (Figure 1).

### Characteristics of the study population and setting

A total of 11 cross-sectional articles were identified, including 11,784 participants, with sample sizes in the range of 244–3,784. Regarding the location of the studies conducted in the region, seven, three, and one were from South Nations Nationalities and People's Region (SNNPR), Oromia, and Amhara, respectively. The prevalence of MWH utilization among women was in the range of 7%–42.5% (11–21, 25). Regarding regional distribution, Oromia showed both the highest and lowest prevalence of utilization (Table 1).

**Table 1:** Summary of study characteristics included in the systematic review and meta-analysis.

Reference	Region	Zone	Study design	Sample size	Prevalence	Status
Gurara et al. (14)	SNNPR	Gamo Goffa	Cross-sectional	814	8.40	Low risk
Kurji et al. (17)	Oromia	Jimma	Cross-sectional	3,784	7.00	Low risk
Selbana et al. (15)	SNNPR	Keffa	Cross-sectional	379	42.50	Low risk
Vermdein et al. (16)	SNNPR	Gurage	Cross-sectional	244	18.30	Low risk
Meshesha et al. (11)	SNNPR	Debub Omo	Cross-sectional	516	16.70	Low risk
Kurji et al. (21)	Oromia	Jimma	Cross-sectional	3,784	28.00	Low risk
Tirunesh (26)	Amhara	Angolela Tera	Cross-sectional	522	24.90	Low risk
Petros (2020)^1^	SNNPR	K/Tambaro	Cross-sectional	495	28.10	Low risk
Getachew et al. (12)	SNNPR	Gurage	Cross-sectional	716	28.20	Low risk
Teshome et al. (19)	Oromia	Arissi	Cross-sectional	530	23.60	Low risk
Kebede and Mihrete (20)	SNNPR	Kefa/Bench Maji	Cross-sectional	Qualitative	Qual.	Low risk

SNNPR, Southern Nations', Nationalities', and People's Region.

### Maternity waiting home utilization

**Figure 2:**
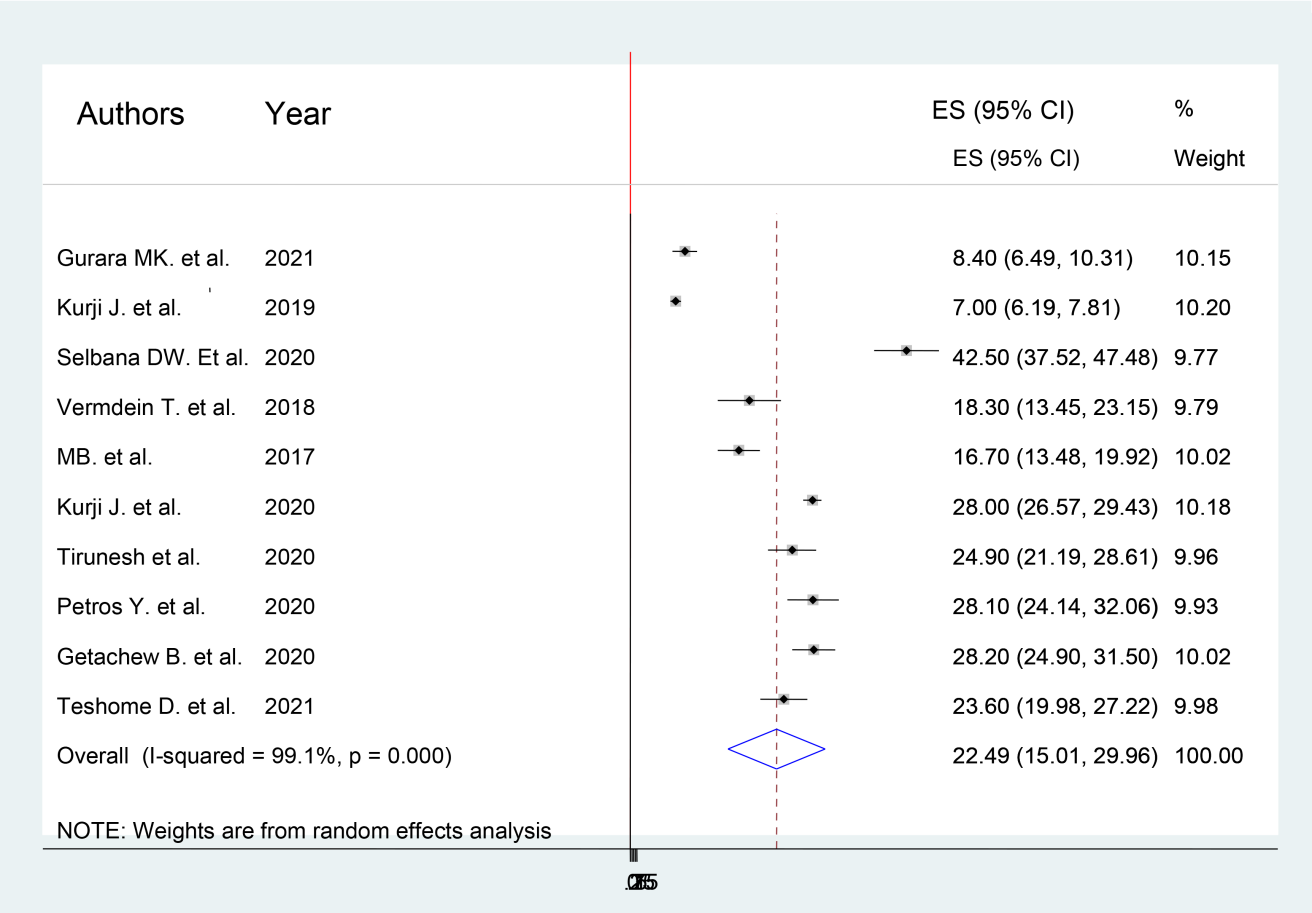
Forest plot of studies on maternity waiting homes utilization in Ethiopia.

The estimated pooled prevalence of women's utilization of maternity waiting homes in Ethiopia was 22.49% (95% CI 15.01%–29.86%) using a random-effects model (*I*^2^ 99.1%, *p* < 0.01) (Figure 2).

### Publication bias analysis

**Figure 3:**
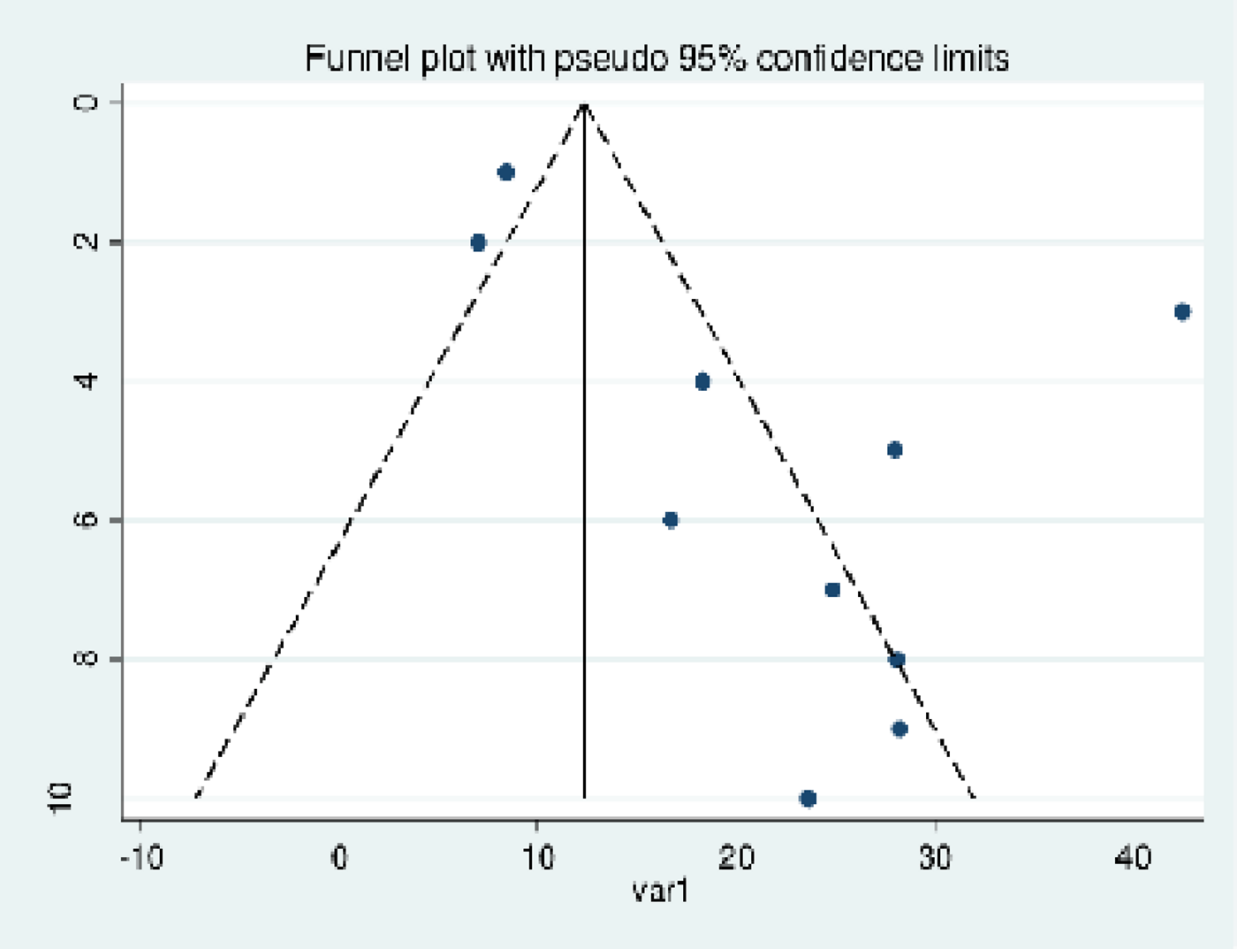
Funnel plot of the 10 studies on women’s utilization of maternity waiting homes in Ethiopia.

The publication bias assessment revealed an asymmetrical distribution in the funnel plot (Figure 3), suggesting the presence of publication bias. Egger's regression test for publication bias was found to be *p* < 0.001, showing a significant publication bias. As a result, a trim-and-fill analysis was conducted to address the publication bias. After one study with a high effect size was removed, nine studies were computed and revealed a pooled prevalence of 19.6% (95% CI 14.00%–25.90%) using a random-effects model. We used a random-effects model for the variation among studies.

### Sensitivity analysis

A sensitivity analysis was conducted to identify any sources of heterogeneity in the pooled prevalence of maternity waiting home utilization. The analysis identified one study with a weighted prevalence of 20.23% (95% CI 8.27%–49.48%). For the remaining studies, the weighted prevalence was in the range of 9.31% (95% CI 4.80%–18.08%) to 11.70% (95% CI 6.21%–22.07%) (Table 2).

**Table 2:** Sensitivity analysis of each study on the utilization of maternity waiting homes in Ethiopia.

S. No	Study omitted	Estimate	95% CI
1.	Gurara et al. (14)	11.71	6.21–22.07
2.	Kurji et al. (17)	20.23	8.27–49.48
3.	Selbana et al. (15)	11.10	6.05–20.34
4.	Vermdein et al. (16)	11.23	6.12–20.59
5.	Meshesha et al. (11)	11.16	6.05–20.59
6.	Kurji et al. (21)	9.31	4.80–18.08
7.	Tirunesh (26)	11.08	6.02–20.38
8.	Petros (2020)^1^	11.08	6.03–20.36
9.	Getachew et al. (12)	10.97	5.95–20.22
10.	Teshome et al. (19)	11.08	6.02–20.40
11.	Combined	11.32	6.20–20.66

### Subgroup analysis

**Figure 4:**
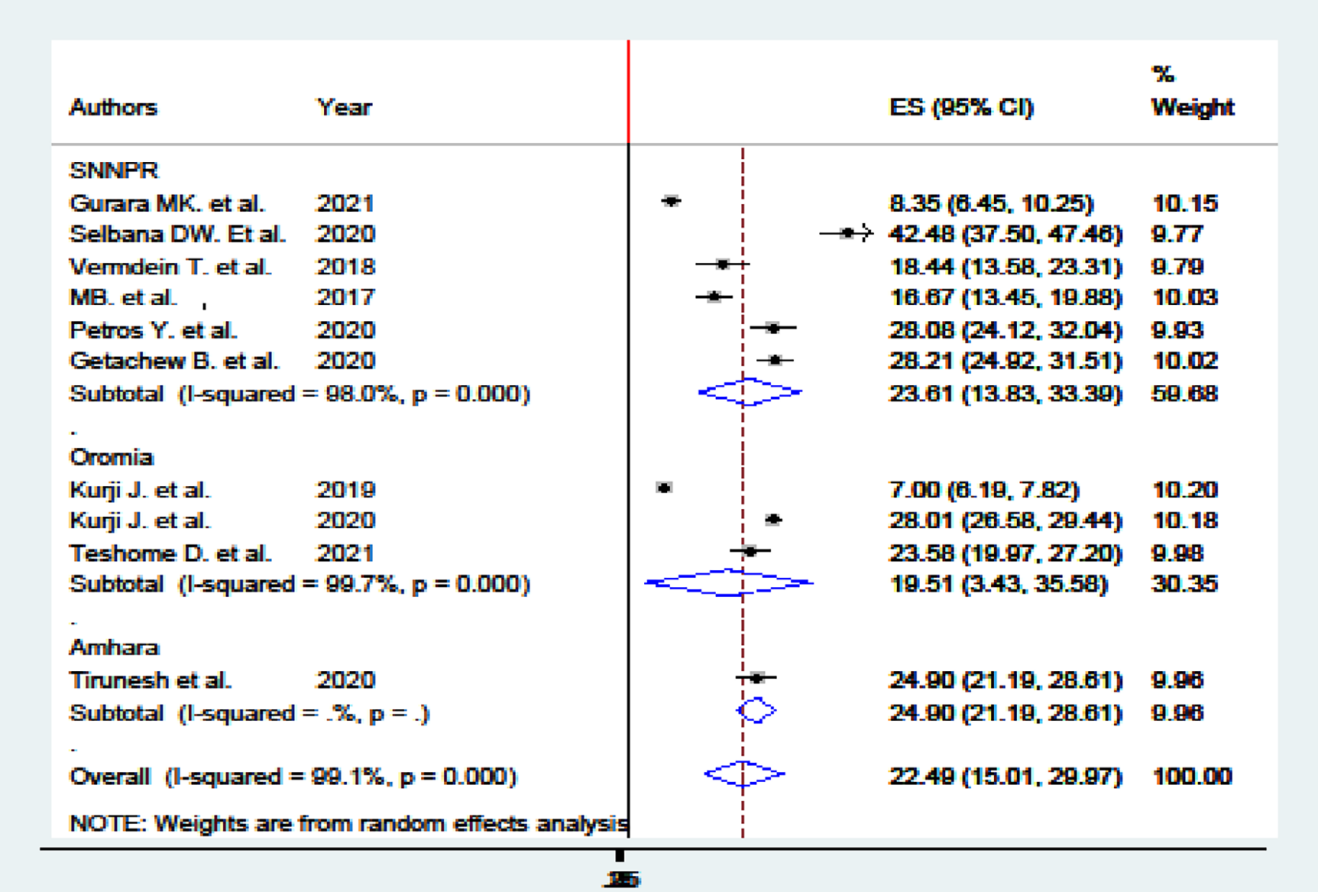
Subgroup analysis on region of studies on utilization of MWH in Ethiopia.

Based on the findings that indicated heterogeneity between studies, a subgroup analysis was carried out for study regions and study period categories. The subgroup analysis by region shows that the pooled prevalence of women's utilization of maternity waiting homes was highest in South Nations, Nationalities, and People's Region at 23.61% (95% CI 13.83%–33.39%) and Oromia at 19.51% (95% CI 3.43%–35.35%). The subgroup analysis based on the year of study estimated that the highest prevalence was in 2020 at 29.99% (95% CI 25.85%–34.14%) and the lowest was in 2019 at 7.00% (95% CI 6.19%–7.82%) (Figure 4).

### Factors associated with the utilization of MWHs

Among the primary studies selected, various factors were significantly associated with women's utilization of MWH and these were pooled together. Four studies identified that women decision-making power (14, 15, 19, 26) and three studies showed that residing within walking distance (≤1 h) (14, 19, 21) had an adjusted odds ratio (AOR) of 1.92 (95% CI 1.61–2.24) and 1.88 (95% CI 1.20–2.56), respectively, which was significantly associated with women's utilization of MWH in Ethiopia. In contrast, two studies showed that access to transport (14, 19) and having a companion during the stay in the MWH (15, 21) had an AOR of 1.92 (95% CI 0.23–3.61) and 0.73 (95% CI −0.43–1.89), respectively, and were not associated with women's utilization of MWH in Ethiopia.

Women with decision-making power were 1.92 times more likely to utilize MWH than those without decision-making power (AOR = 1.92, 95% CI 1.61–2.24). Women who resided within a walking distance of ≤1 h to the nearest MWH were 1.88 times more likely to utilize MWHs than those who lived further away (AOR = 1.88, 95% CI 1.20–2.56).

**Figure 5:**
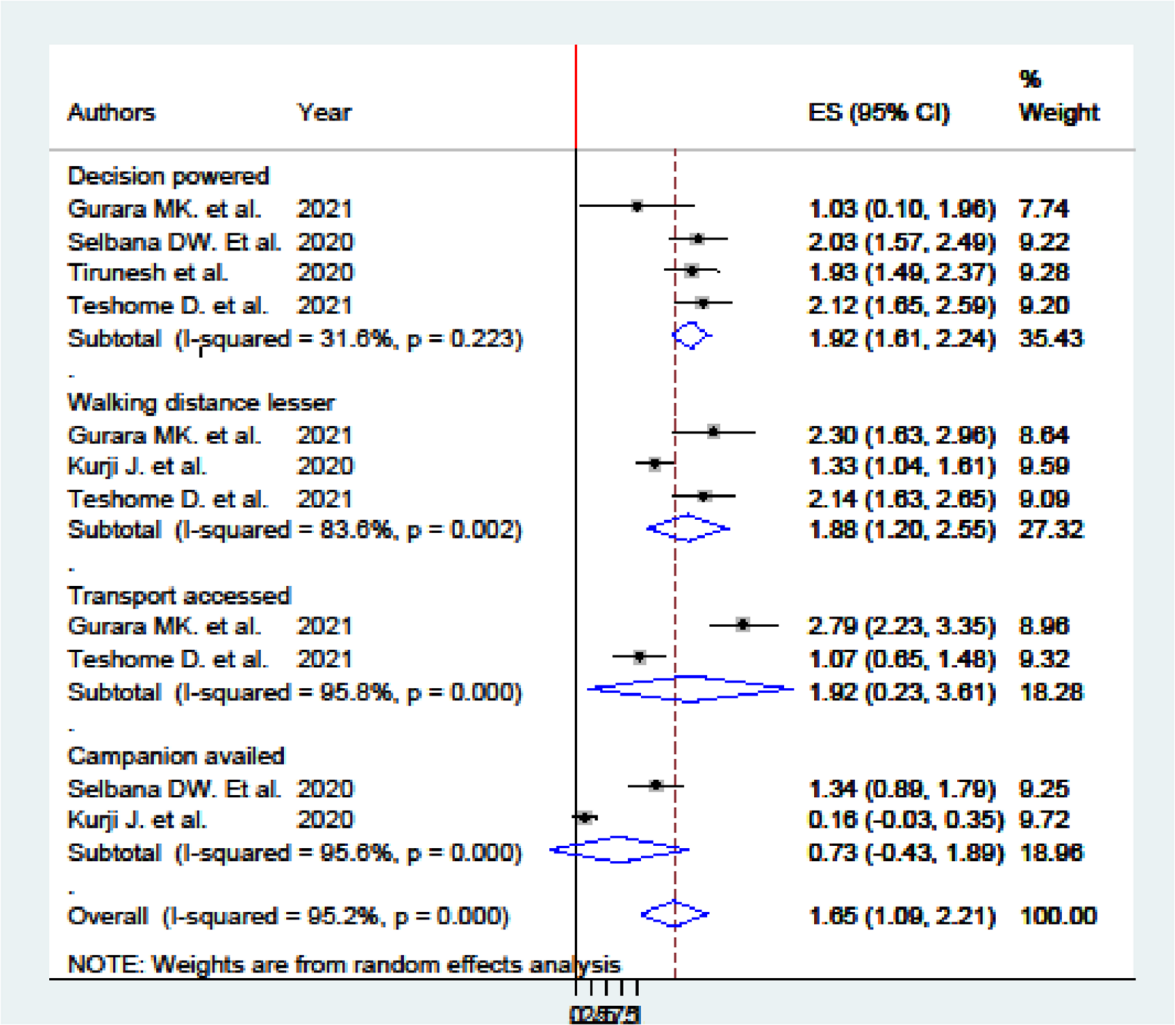
Forest plot of studies: association of factors with maternity waiting home utilization in Ethiopia.

Based on the meta-analysis of two studies, women with access to transportation to the maternity waiting home were 1.92 times higher to utilize the MWH than those who had no access to transportation to it (AOR = 1.92, 95% CI; 0.23–3.61). On the other hand, the estimated odds of women who had a companion showed no significant association with MWH utilization compared with their counterparts (AOR = 0.73, 95% CI −0.43 to 1.89) (Figure 5).

In addition, the findings of the qualitative study reviewed revealed factors hindering utilization, including lower wealth status of the household, poor quality of services and structure of facilities in maternity waiting homes, and women's poor perception of maternity waiting homes (20).

## Discussion

Worldwide, evidence-based maternal healthcare implementations are vital for reducing maternal mortality and morbidity in middle- and low-income countries, including Ethiopia. Maternity waiting home service utilization is a key approach to accessing care during childbirth and can help alleviate maternal death. It brings expectant mothers closer to the appropriate health facilities to ensure access to emergency obstetric and newborn care. This could not be achieved without adequate evidence of MWH utilization and its affecting factors.

In this systematic review and meta-analysis, the estimated pooled prevalence of women's utilization of MWH in Ethiopia was 22.49% (95% CI 15.01%–29.86%). This finding is significantly lower than the nationally expected level of 100% utilization of MWHs where they are established and functional (24). This might be due to the MWH strategy being a new initiative, influenced by factors from women, social, and health facility sides. The result of this systematic review and meta-analysis is also lower than the individual studies conducted in Zimbabwe (33.32%) (27) and Cuba (30.00%) (28). The difference could be due to this review including results from several regions in Ethiopia, whereas the studies from Zimbabwe and Cuba were individual studies. In addition, variations in sociodemographic and socioeconomic characteristics may explain these differences as healthcare-seeking behavior might be affected by societal values, cultural perceptions of healthcare needs, income, and access to healthcare. The differences in sample size between the studies might also contribute; larger sample sizes, which are closer to the total population, may decrease the proportion parameter.

Concerning the subgroup analysis results by region and year of publication, the highest level of utilization of maternity waiting homes among women was reported in SNNPR, with the highest utilization also observed in studies published in 2020. This dissimilarity might be due to the regions and study years with higher utilization having a larger number of studies in the subgroup compared to their counterparts. This suggests that there is significantly high utilization of maternity waiting homes in these regions and years.

In addition to analyzing the pooled prevalence of MWH utilization, the meta-analysis was applied to identify factors associated with MWH utilization. The ability of women to independently decide to use a MWH was positively associated with its utilization. This may be because being able to make decisions regarding their healthcare increases the likelihood of women utilizing a maternity waiting home rather than seeking permission from others. This finding is consistent with the qualitative studies reviewed (20) and other studies conducted in other regions (9, 25, 26, 29–33).

This systematic review and meta-analysis found that women living within a short walking distance to the nearest maternity waiting home were significantly more likely to utilize the service. This could be because the proximity of a health facility increases a woman’s confidence during her stay in the MWH. It also allows her to visit her home and return more easily. However, this finding does not support the maternity waiting home strategy, which is primarily aimed at addressing maternal mortality caused by delays in accessing care. Maternity waiting homes are expected to serve women from areas that are difficult to access (34).

According to this meta-analysis, women who had a companion with them at the MWH showed no statistically significant association with utilization. This finding differs from other studies. This discrepancy might be due to the sociodemographic differences in the study populations, as well as variations in perception, value, and culture, especially given that Ethiopia is a diverse country with more than 89 ethnic groups. However, women without a companion might feel vulnerable and seek support from someone close to them when away from home. Similarly, women might lose confidence in staying outside their homes, leading to a fear of helplessness (35–37).

In addition, the findings from individual studies and the qualitative study reviewed revealed several factors hindering the utilization of maternity waiting homes, including lower household wealth, poor quality in the services and structure of the facilities, and women's negative perceptions of maternity waiting homes. Similarly, other studies identified negative cultural attitudes, food shortages at MWHs, and a lack of privacy contributing to the low utilization of MWHs (34–40).

### Limitations of the review

Although this review has identified new information, it has some limitations. Since the studies selected for review were cross-sectional, they have less power to control for confounding factors. In addition, the studies included in the review were not representative of regions of the country, which may affect the accuracy of the findings in reflecting the national status of the issue. The review also excluded non-English language studies, which may have influenced our results. Furthermore, the review faced a shortage of related studies conducted in Ethiopia or other African countries, which limited the discussion on the findings.

## Conclusion

The pooled prevalence of women's utilization of MWH was much lower than the expected target. Factors identified as predictors of women's utilization of MWHs include decision-making status and walking distance of ≤1 h to the nearest care facility. The authors recommend that the health sector administrators at the woreda and zonal levels work on both the consumer and healthcare system sides, particularly by focusing on MWH advocacy at the community level and improving MWH facilities. Finally, the authors suggest conducting further studies to generate additional evidence.

## Data Availability

The original contributions presented in the study are included in the article/Supplementary Material, further inquiries can be directed to the corresponding author.

## Footnotes

^1^Peteros Y. Utilization of pregnant mothers waiting area and factors associated among mothers at Damboya district Kembata Tembaro Zone, South Ethiopia (thesis). (2020), unpublished. Available online at: http://10.140.5.162//handle/123456789/4215 (accessed May 28, 2021).
